# Integrated Co-extraction Protocol for Transcriptomic and H NMR Metabolomic Analysis of Multi-species Biofilms

**DOI:** 10.21769/BioProtoc.5237

**Published:** 2025-03-05

**Authors:** Anaïs Séguéla, Oriane Della-Negra, Roselyne Gautier, Jérôme Hamelin, Kim Milferstedt, Rémi Servien, Marie-Ange Teste, Cécile Canlet

**Affiliations:** 1 Toxalim (Research Centre in Food Toxicology), Toulouse University, INRAE, ENVT, INP-Purpan, Toulouse, France; 2 MetaboHUB-MetaToul, National Infrastructure of Metabolomics and Fluxomics, Toulouse, France; 3 INRAE, Univ. Montpellier, LBE, Narbonne, France; 4 TBI, Université de Toulouse, CNRS, INRAE, INSA, Toulouse, France; 5 IBISBA-GeT-Biopuces, Toulouse, France

**Keywords:** Biofilm, NMR, RNA, Metabolomics, (meta)transcriptomic, Oxygenic photogranules, Multi-omics

## Abstract

Capturing produced, consumed, or exchanged metabolites (*metabolomics*) and the result of gene expression (*transcriptomics*) require the extraction of metabolites and RNA. Multi-omics approaches and, notably, the combination of metabolomics and transcriptomic analyses are required for understanding the functional changes and adaptation of microorganisms to different physico-chemical and environmental conditions. A protocol was developed to extract total RNA and metabolites from less than 6 mg of a kind of phototrophic biofilm: oxygenic photogranules. These granules are aggregates of several hundred micrometers up to several millimeters. They harbor heterotrophic bacteria and phototrophs. After a common step for cell disruption by bead-beating, a part of the volume was recovered for RNA extraction, and the other half was used for the methanol- and dichloromethane-based extraction of metabolites. The solvents enabled the separation of two phases (aqueous and lipid) containing hydrophilic and lipophilic metabolites, respectively. The ^1^H nuclear magnetic resonance (NMR) analysis of these extracts produced spectra that contained over a hundred signals with a signal-to-noise ratio higher than 10. The quality of the spectra enabled the identification of dozens of metabolites per sample. Total RNA was purified using a commercially available kit, yielding sufficient concentration and quality for metatranscriptomic analysis. This novel method enables the co-extraction of RNA and metabolites from the same sample, as opposed to the parallel extraction from two samples. Using the same sample for both extractions is particularly advantageous when working with inherently heterogeneous complex biofilm. In heterogeneous systems, differences between samples may be substantial. The co-extraction will enable a holistic analysis of the metabolomics and metatranscriptomics data generated, minimizing experimental biases, including technical variations and, notably, biological variability. As a result, it will ensure more robust multi-omics analyses, particularly by improving the correlation between metabolic changes and transcript modifications.

Key features

• Co-extraction of metabolites and total RNA from 6 mg of dry biomass of phototrophic biofilms, notably oxygenic photogranules.

• Biphasic metabolome extraction for the characterization of hydrophilic and lipophilic metabolites using ^1^H NMR.

• Total RNA extraction with sufficient quality and quantity for analysis of (meta)transcriptome.

Graphical overview

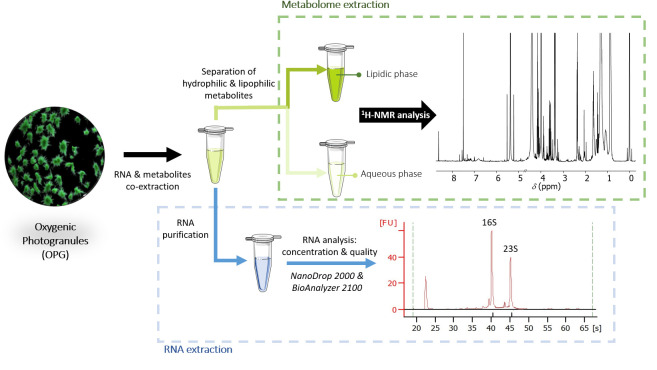

Metabolomic and RNA co-extraction from oxygenic photogranules

## Background

In contrast to suspended individual cells, biofilms are spatialized microbial structures composed of diverse interacting microbial populations. Gradients created by energy and mass transfer as well as the production and consumption of metabolites in the biofilm matrix generate a wide range of physiological states of the resident microbial community. The biofilm environment implies genetic and phenotypic heterogeneity, with distinct metabolic pathways and varied responses to stress and environmental conditions along the gradients. These sources of heterogeneity affect functional and metabolic responses in space and time [1,2].

Elucidating the physiologies of biofilm-associated microorganisms by capturing metabolite production and gene expression is key to deciphering how multi-species biofilms adapt to changing environments as a community. Various methods are currently available to study the dynamics of biofilms, including metabolomics [3–5] and transcriptomic [6,7] approaches. Multi-omics approaches, combining metatranscriptomics and metabolomics through separate metabolome and RNA extraction procedures, have been applied to the study of some biofilms [8–10]. However, to the best of our knowledge, a protocol that enables the simultaneous extraction of both metabolites and RNA from biofilms has not yet been described.

Metabolomics focuses on the detection of metabolites (for example, produced during biofilm formation) and characterizes the state of the metabolome at a given time. In this way, metabolomics provides a view of the general physiological state of the biofilm community in a given condition. Metabolite profiles are functional signatures that can indicate an actual phenotype [5]. Nuclear magnetic resonance (NMR) spectroscopy is increasingly used in metabolomics and biofilm characterization due to the simplicity of measurements compared with other methods such as mass spectrometry. Compared to mass spectrometry, NMR is less sensitive, focuses on the major changes in metabolic pathways, and is not sufficient to profile all the metabolites and identify all functional changes [11]. However, NMR offers numerous advantages as it generally includes a simplified sample pre-treatment and allows immediate (semi) quantification of metabolites and robust metabolite assignment [12]. It is independent of ionization efficiency and can detect a wide range of metabolites, including those that ionize poorly in mass spectrometry, such as sugars, organic acids, and some lipids usually found in biofilms. Thus, this method is particularly adapted for characterizing and identifying compositional changes in biofilms.

Metatranscriptomic analysis enables the study of the total mRNA transcripts and reveals the global gene expression profiles and changes. It can give insights into the functional components activated or inhibited during biofilm growth or decay. It can also be used to monitor biofilm dynamics in response to changes in physico-chemical or environmental conditions [13].

Currently, most omics studies are conducted separately, resulting in a partial picture of the state of the biological system. If conducted together, they generally result in distinct extraction procedures [8–10]. The integration of metabolomic and metatranscriptomic approaches offers the potential to uncover novel correlations between metabolites and transcripts. However, in biofilms, their inherent structural, microbiological, and functional heterogeneity amplifies the variability of results when multiple extractions are performed. To minimize experimental biases, including technical variations and biological variability, a protocol that allows the simultaneous extraction of RNA and metabolites is essential. Co-extraction enhances the robustness of the results by ensuring that metabolic and transcriptomic changes can be directly correlated within the same sample, providing a more reliable and comprehensive understanding of biofilm dynamics.

In this study, oxygenic photogranules (OPGs) were used as a model biofilm system to develop a co-extraction protocol for metabolites and RNA, enabling NMR-based metabolomics and metatranscriptomic analyses. OPGs are three-dimensional aggregates with spatial and compositional (microbial communities and metabolites) heterogeneity. They contain a syntrophic community composed of heterotrophic and phototrophic microorganisms (eukaryotic algae and cyanobacteria) [14]. They are obtained in controlled conditions from activated sludge and are currently under study for wastewater treatment [15].

## Materials and reagents

**Biological materials**

**Figure 1.**
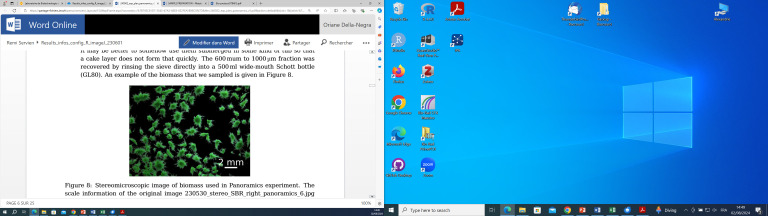
Stereomicroscopic image of photogranules obtained after the sieving step

Metabolites and RNA were co-extracted from oxygenic photogranules (OPGs) (Figure 1). Photogranules are microbial aggregates in the size range of several hundreds of micrometers up to several millimeters. They harbor a microbial community primarily composed of a phototrophic population, often dominated by one or two filamentous cyanobacteria of the order Oscillatoriales and a taxonomically and functionally diverse population of heterotrophic bacteria. Photogranules were produced using previously described protocols [14,15]. Briefly, OPGs were grown in an open sequencing batch reactor operated at an average temperature of 25 °C with overhead stirring of 110 rpm. LED panels provided white light at 4,000 K, yielding 96 μmol/m^2^/s photosynthetically active radiation at the outside of the vertical surfaces of the reactor. The reactor had a working volume of 4 L. The sequencing batch cycle had a duration of 3 h. Two liters of bulk phase were replaced in every cycle with a synthetic medium at a carbon concentration of 33 mg/L as chemical oxygen demand, resulting in a hydraulic retention time of 6 h. The synthetic medium contained 0.5 mM sodium acetate, 6 μM KH_2_PO_4_, 5 μM Na_2_HPO_4_, 0.13 mM (NH_4_)_2_SO_4_, 1.1 μM EDTA·2H_2_O, 0.9 nM Na_2_MoO_4_·2H_2_O, 6 nM FeSO_4_·7H_2_O, 0.042 μM H_3_BO_3_, 3 nM ZnSO_4_·7H_2_O, 2 nM MnCl_2_, 7 nM CoCl_2_·6H_2_O, 0.01 μM CuSO_4_·5H_2_O, 0.2 μM FeCl_3_, and 0.9 nM NiCl_2_·6H_2_O.

**Reagents**

1. Ethanol absolute (Fisher, catalog number: 10048291)

2. Methanol (Fisher, catalog number: 10499560)

3. Dichloromethane (Fisher, catalog number: 10626642)

4. Disodium hydrogen phosphate anhydrous (Na_2_HPO_4_) (Sigma-Aldrich, catalog number: 71640)

5. Potassium phosphate monobasic (KH_2_PO_4_) (Sigma-Aldrich, catalog number: P5655)

6. 3-(Trimethylsilyl)propionic acid-*d4* sodium salt (TSP) (Sigma-Aldrich, catalog number: 269913)

7. Deuterium oxide (D_2_O) (Sigma-Aldrich, catalog number: 151882)

8. Methanol-*d4* (CD_3_OD) (Sigma-Aldrich, catalog number: 151947)

9. Chloroform-*d* (CDCl_3_) + 0.03% trimethylsilane (TMS) (Sigma-Aldrich, catalog number: 343803)

10. RNeasy Mini kit (Qiagen, catalog number: 74104)

11. Qubit 1× dsDNA HS Assay kit (Thermo Fisher Scientific, Invitrogen^TM^, catalog number: Q33230)

12. DNA-free^TM^ DNA Removal kit (Thermo Fisher Scientific, Invitrogen^TM^, catalog number: AM1906)

12. RNaseZap (Thermo Fisher Scientific, Invitrogen^TM^, catalog number: AM9780)

14. RNA 6000 Nano kit (Agilent, catalog number: 5067-11511)

Solutions

1. 70% ethanol solution (see Recipes)

2. Buffer phosphate solution (see Recipes)

**Recipes**

**1. 70% ethanol solution**

**Table:** 

Reagent	Final concentration	Quantity or Volume
Ethanol absolute	70%	700 mL
MilliQ water (H_2_O)	n/a	300 mL
Total	n/a	1000 mL

**2. Buffer phosphate solution**

Weigh all components mentioned below and dissolve in 100 mL of deuterated water. Measure and adjust the pH to 7.0 with 5 M HCl.

**Table:** 

Reagent	Final concentration	Quantity or Volume
Na_2_HPO_4_	200 mM	2.885 g
KH_2_PO_4_	40 mM	595 mg
TSP	0.2 mM	3.45 mg
D_2_O	n/a	100 mL
Total	n/a	100 mL

**Laboratory supplies**

**A. Sampling**

1. Lysing matrix E (MP Biomedicals, catalog number: 1169140-CF)

2. Sieves (200 mm DIA × 50 mm ISO 3310–1); 0.6 mm (Retsch, catalog number: 60131000600, 23208274) and 1.0 mm (Fisher Scientific, catalog number: 10536222, 7156588)

3. Liquid nitrogen

4. Dry ice

**B. Co-extractions**

1. QIAshredder (Qiagen, catalog number: 79654)

3. 1.5 mL microtubes (e.g., Eppendorf, catalog number: 0030125150)

4. Filter tips 1,250, 200, and 20 μL (e.g., Clearline, catalog numbers: 713119, 713117, 713115)

5. 2, 10, 20, 200, and 1,000 μL pipettes (e.g., Clearline or Gilson)

**C. NMR analysis**

1. 1.75 mL Eppendorf tubes (Dutscher, Greiners, catalog number: 2710757)

2. Pipette tips 1,250 and 200 μL (Dutscher, Clearline, catalog number: 713113, 713111)

3. Pasteur pipette (Dutscher, catalog number: 2517260)

4. NMR tubes 3 mm including caps (Bruker, catalog number: Z112272)

5. Cap sealing balls (Bruker, catalog number: Z147554)

## Equipment

1. Laboratory fume hood (e.g., Waldner, model: MC6 1500 mm)

2. FastPrep-24^TM^ 5G bead beating grinder and lysis system (MP Biomedicals, catalog number: 116005500)

3. Microcentrifuge (e.g., Eppendorf, model: 5425)

4. Refrigerated centrifuge (e.g., Sartorius, model Centrisart A-14C; Mikkro 220R, Dutscher, catalog number: 472328)

5. Multivortex Multireax (Heidolph, Dutscher, catalog number: 498446)

6. Bioanalyzer 2100 (Agilent, catalog number: G2939BA)

7. Nanodrop ND2000 (Thermo Scientific, catalog number: ND-2000)

8. Qubit 4 fluorometer (Thermo Scientific, catalog number: Q33238)

9. -20 °C freezer

10. -80 °C freezer

11. pH meter HI 3221 (Hanna Instruments, Dutscher, catalog number: 054585)

12. SpeedVac SPD 300 DDA (Dutscher, Fischer, catalog numbers: 22880, 228324, 228731)

13. Sample preparation robot (Tecan, model: Fluent 780)

## Software and datasets

1. TopSpin (Bruker Biospin, Germany, V3.6.4, February 2023)

2. R software (https://cran.r-project.org/, V4.2.2, November 2022)

3. ASICS R package (V2.20.1)

4. 2100 expert software (Agilent, V B.02.10.764, July 2018)

5. Nanodrop 2000 software (V1.6.198, August 2014)

## Procedure

**A. Experimental setup and sampling**

1. Sort the oxygenic photogranules by size using 0.6 and 1.0 mm mesh sieves.

2. Prepare batches of 50 small OPGs (approximately 6 mg of dry biomass for 50 OPGs) in 2 mL screw-cap tubes containing 500 μL of tap water.

*Notes:*

*1. The minimum quantity of biomass required for the validity of the method may vary. It is therefore recommended to test the co-extraction method on a gradient of biofilm biomass. The smallest usable mass should be retained for the actual co-extraction.*

*2. At this stage, you can expose your photogranules or biofilms to varying experimental conditions (light, carbon supply, etc.). We recommend having at least eight replicates per condition to ensure robust statistical analyses of the data.*

3. Twenty-four hours before sampling, store the tubes containing the lysing matrix E at -80 °C.

*Note: The lysing matrix E, used for OPG grinding, contains 1.4 mm ceramic spheres, 0.1 mm silica spheres, and one 4 mm glass bead.*

4. Immediately after sampling, freeze the biomass in liquid nitrogen.

*Notes:*

*1. It is important to make sure that the biomass is at the bottom of the vials so that the buffer can be added easily for further treatment of the vials.*

*2. If the co-extraction is not performed just after sampling, tubes can be stored at -80 °C.*

*3. Here, RNA and metabolites were extracted from oxygenic photogranules, including the interstitial liquid phase and the culture media. However, this protocol could be adapted to dry pellets.*

***Critical***: *The initial freezing at a very low temperature is mandatory to instantaneously stop metabolic and transcriptomic activity, maintain RNA and metabolome integrity, and avoid loss of information and erroneous results. Here, we recommend the use of liquid nitrogen that meets the temperature requirements and ensures efficient temperature transfer via an optimum contact surface. Once the sample is frozen, it can be stored at -80 °C.*

5. Addition of the beads to the samples:

a. Remove the lysing matrix E from the -80 °C freezer and store the tubes on dry ice.

b. If the samples were stored in a -80 °C freezer, remove the samples and put them into a cryo box containing a bed of dry ice.

c. Open the sample tubes and discard the caps.

d. Add the cooled lysing matrix E to the samples.

e. Use the caps from the lysing matrix tubes to close the tubes now containing the photogranules and lysing matrix E. This simplifies tube handling.

*Note: Switching the caps simplifies the workflow. The O-ring of the original caps sometimes detaches when opening the tubes. Putting them back in place can be cumbersome. Using the new caps avoids this issue.*

6. Return the samples to -80 °C until co-extraction.

*Note: We recommend storing the samples at -80 °C for no longer than 12 months to avoid any degradation of the metabolome or RNA.*

**B. Co-extraction**

1. Put the samples in ice and add 600 μL of buffer RLT (from RNeasy Mini kit) to the tubes (see General Note 1).

***Critical:*** *To avoid breaking the cold chain, it is important to perform this step on an ice bed.*

2. Grind the samples with the FastPrep for 20 s at 6 m/s to lyse cells.

3. Place the samples in the ice bed for 1 min.

4. Repeat the grinding step.

*Notes:*

*1. Depending on the matrix, the grinding step must be optimized. In photogranules, an indicator of successful lysis of the phototrophs is the release of chlorophyll into the liquid, yielding a bright green supernatant. To check that non-phototrophic cells have been properly lysed, the extracted RNA (or DNA) must be quantified.*

*2. If the sample is not ground enough, add another cycle. Between each cycle, put the sample on the ice bed for 1 min to avoid overheating.*

5. Centrifuge at 16,000× *g* for 2 min at room temperature to allow the large particles to settle.

6. At room temperature, collect the solution and transfer it to the QIAshredder spin column.

*Note: Low temperature can alter the column, so working at room temperature is recommended. Preliminary tests showed a lower RNA extraction yield at 4 °C.*

7. Centrifuge at 16,000× *g* for 2 min at room temperature to remove microbeads and particles.

8. Split the eluate in two:

a. 300 μL for the RNA extraction step (section E), without resuspending the particles collected at the bottom of the column.

b. 450 μL of the remaining volume for metabolite extraction (section C).

*Note: The volume may vary depending on the sample but is limited by the capacity of the QIAshredder spin column (<750 μL).*

**C. Metabolite extraction**

*Note: For the following steps, it is advisable to place samples on an ice bed to avoid any degradation during extraction and to improve stability.*

1. Add 400 μL of methanol to the tube with the remaining volume and pellet from step B.

***Caution:*** *Perform this step in a fume hood because of the toxicity of methanol.*

2. Vortex for 2 min with a multivortex.

3. Add 200 μL of dichloromethane and vortex for 2 min.

***Caution:*** *Perform this step in a fume hood because of the toxicity of dichloromethane.*

4. Vortex for 2 min with a multivortex.

5. Repeat steps C3 and C4.

6. Place the sample on ice for 15 min at 4 °C to allow the two phases to separate.

7. Centrifuge at 2,200× *g* for 15 min at 4 °C in a refrigerated centrifuge.

8. Separate the two phases obtained. The aqueous phase is above the organic phase:

Aqueous phase: collect 700 μL of this phase for each sample.

Organic phase: collect 200 μL of this phase for each sample.

*Notes:*

*1. The volume of the organic phase is approximately 400 μL; however, to avoid accidentally taking any of the aqueous phase or the pellet, collect only 200 μL of organic phase.*

*2. Take the same volume for each sample for repeatability.*

***Caution:*** *Perform these steps under a fume hood.*

9. At this point, samples can be stored at -80 °C until the evaporation step.

*Note: Samples should not be stored for longer than 3 months to avoid any degradation.*

10. Evaporation of the different phases:

a. Aqueous phase:

i. Remove the sample from the freezer and thaw it.

ii. Evaporate the aqueous phase using a SpeedVac: no temperature, manual program, ramp 3.

*Note: To avoid contamination, the Eppendorf tube should not be more than half full. If the volume is too high, split the sample into two tubes. In our case, we proceeded as follows:*

*a. Take 400 μL of the aqueous phase, transfer them to another Eppendorf tube, and evaporate them using the same parameters as in step C9a-ii. Store the Eppendorf tube containing the residual solid at 4 °C.*

*b. Once the first tube is evaporated, transfer the remaining 300 μL of the aqueous phase in this tube and evaporate it.*

iii. Store the samples at -80 °C.

*Note: Samples should be stored for no longer than 3 months to avoid any degradation.*

b. Organic phase: Evaporate the organic phase under nitrogen flow at room temperature over a minimum of 2 h.

***Caution:*** *Perform this step in a fume hood.*

*Note: Once dry, organic samples may have stability problems and can no longer be stored at -80 °C. We therefore recommend transferring them into NMR tubes for immediate analysis.*

**D. NMR tube preparation**

1. Aqueous phase:

a. Prepare the deuterated buffer phosphate solution (see Recipe 2).

b. Using the Tecan Robot, add 270 μL of deuterated buffer to each dry sample and mix with the needles.

*Note: In the absence of a Tecan Robot, manually add 270 μL of buffer and vortex the samples using a multivortex.*

c. Centrifuge at 1,200× *g* for 15 min at 4 °C with a refrigerated centrifuge.

d. With the Tecan Robot, take 25 μL of each sample and pool the aliquots in a 15 mL centrifuge tube to perform quality control.

e. Transfer 200 μL of the samples to NMR tubes of 3 mm diameter.

*Note: If you do not have a Tecan Robot, perform steps D1d and D1e manually with the same volumes.*

f. Fill the openings of the NMR tubes with beads.

*Note: If you do not have a Tecan Robot, close the NMR tubes with a suitable cap.*

2. Organic phase:

***Caution:*** *Perform the following steps in a fume hood.*

a. Prepare a solution of deuterated chloroform with 0.03% TMS and deuterated methanol (2:1 in volume).

b. Add 270 μL of this solution to each sample using a Pasteur pipette.

c. Take 25 μL of each sample and put them into a glass tube for quality control.

d. Transfer 200 μL of the samples into NMR tubes of 3 mm diameter.

e. Fill the openings of the NMR tubes with cap sealing balls (see D1f).

*Note: Close the NMR tubes with a suitable cap*.

**E. RNA extraction**

1. Take the 300 μL of RNA extraction eluate (see step B8a) and add 300 μL of 70% ethanol (see Recipes 1). Mix by pipetting.

2. Transfer approximately 600 μL into the RNeasy Spin column.

*Note: Always keep the spin columns at room temperature*.

3. Centrifuge at maximum speed (approximately 16,000× *g*) for 1 min. Discard the eluate.

4. Add 700 μL of RW1 buffer (from RNeasy Mini kit).

5. Centrifuge at maximum speed (approximately 16,000× *g*) for 1 min. Discard the eluate.

6. Add 700 μL of RPE buffer (from RNeasy Mini kit).

7. Centrifuge at maximum speed (approximately 16,000× *g*) for 1 min. Discard the eluate.

8. Add 500 μL of RPE buffer.

9. Centrifuge at maximum speed (approximately 16,000× *g*) for 1 min. Discard the eluate.

10. Centrifuge at maximum speed (approximately 16,000× *g*) for 2 min to eliminate all the RPE buffer.

11. Place the column in a new Eppendorf tube (final tube).

12. Add 35 μL of water to the center of the column.

13. Centrifuge at maximum speed (approximately 16,000× *g*) for 2 min.

14. Take the eluate and place it again on the column.

15. Centrifuge at maximum speed (approximately 16,000× *g*) for 2 min.

16. Keep an aliquot (5 μL) for quantification and quality control of your sample (e.g., Nanodrop or Qubit 4 fluorometer & BioAnalyzer) to avoid freeze-thaw cycles of your RNA sample and store the rest at -20 °C (see Data analysis B).

## Data analysis

**A. NMR analysis**

^1^H NMR spectra were obtained with a 300 K Bruker Avance III HD 600 MHz NMR spectrometer (Bruker Biospin, Rheinstetten, Germany), operating at 600.13 MHz for ^1^H resonance frequency, equipped with a 5 mm ^1^H-^13^C-^15^N-^31^P cryoprobe attached to a cryo platform (the preamplifier unit). Probe tuning and matching, locking, shims tuning, pulse duration (90°), and gain computation were automatically performed for each sample. For aqueous samples, ^1^H NMR spectra were acquired using the NOESY-presat ^1^H experiment (noesypr1d: sequence used for suppression of the water signal), whereas for the organic phase, ^1^H NMR spectra were acquired using a standard ^1^H experiment (sequence: zg). A total of 256 transients were collected into 64 k data points using a spectral width of 20 ppm, a relaxation delay of 5 s, and an acquisition time of 2.72 s. Prior to Fourier transformation, an exponential line broadening function of 0.3 Hz was applied to the free induction decays (FID). All NMR spectra were phase- and baseline-corrected and referenced to the chemical shift of TSP or TMS (0 ppm) using the Topspin software. Data analysis was carried out using R version V 4.2.2 [16] using the ASICS package version V2.20.1 [17]. This package is based on a statistical linear model to identify and quantify metabolites in complex NMR spectra using a library containing pure metabolite spectra. The processing method includes automatic baseline correction, normalization, alignment, and exclusion of undesired areas (D_2_O 4.5–5.1 ppm, CD_3_OD 3.33–3.38 ppm, CDCl_3_ 7.2–7.3 ppm, dichloromethane 4.88–5.31 ppm). ^1^H NMR spectra were cleaned by setting a noise threshold of 0.1 for NMR spectra from the aqueous phase and 0.02 for those from the organic phase.

**B. RNA quality control**

1. Measurement of RNAs concentration

Total RNA concentration was quantified using Nanodrop 2000. In order to achieve the ribodepletion for the metatranscriptomics experiments, the concentration of extracted RNA must be high enough (in our case, around 100 ng/μL). As the Nanodrop measures total nucleic acids (DNA and RNA), it is essential to know the real concentration of total RNAs. Therefore, we measured DNA contamination with the Qubit 4 fluorometer using 1 μL of extracted RNAs in 199 μL of Qubit 1× dsDNA HS Assay Kit buffer.

**Figure 2.**
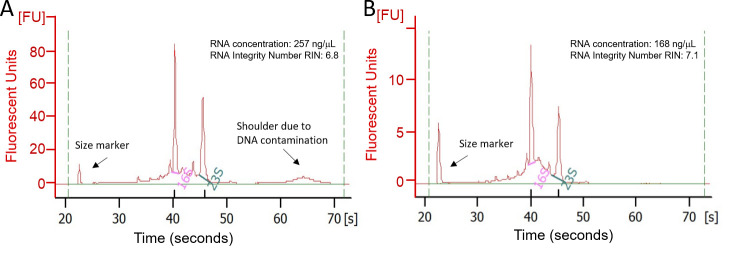
Comparison of RNA profiles of DNase-treated and non-treated samples obtained using the Qubit 4 fluorometer. A. Before DNase treatment. B. After DNase treatment.

*Note: If the DNA contamination is higher than 10%, it will be necessary to treat the total RNAs with DNase (see Figure 2).*

2. Treatment of RNA eluate with DNase

a. Add 2 μL of DNase buffer and 1 μL of DNase (contained in the DNA-free^TM^ DNA Removal kit) to 20 μL of eluted mRNA (2–2.5 μg of total RNA).

b. Incubate for 20 min at 37 °C.

c. Add 2.3 μL of the inactivation reagent contained in the DNA-free^TM^ DNA Removal kit and incubate for 2 min at room temperature with occasional mixing.

d. Centrifuge at maximum speed (approximately 16,000× *g*) for 2 min and transfer the supernatant to a clean tube.

*Note: Perform reactions in small tubes (0.2 or 0.5 mL) to simplify the removal of supernatant after treatment with the DNase inactivation reagent.*

e. After DNase treatment, measure again the RNA concentration with the Qubit 4 fluorometer as described above. The measured value will be used for the remaining experiments.

3. Measurement of quality and integrity of the extracted RNA

The quality and integrity of the extracted RNA were checked using the Bioanalyzer 2100 and Bioanalyzer RNA 6000 Nano kit. RNAs were denatured for 2 min at 70 °C and put on ice; 1 μL was used according to the manufacturer's recommendations.

## Validation of protocol

This protocol was tested on a set of 200 samples, demonstrating its robustness and reproducibility, obtaining sufficient concentrations of metabolites and RNA for subsequent metabolomic and metatranscriptomic analyses, while complying with the quality criteria for NMR spectra and RNA.

**A. NMR analysis**

**Figure 3.**
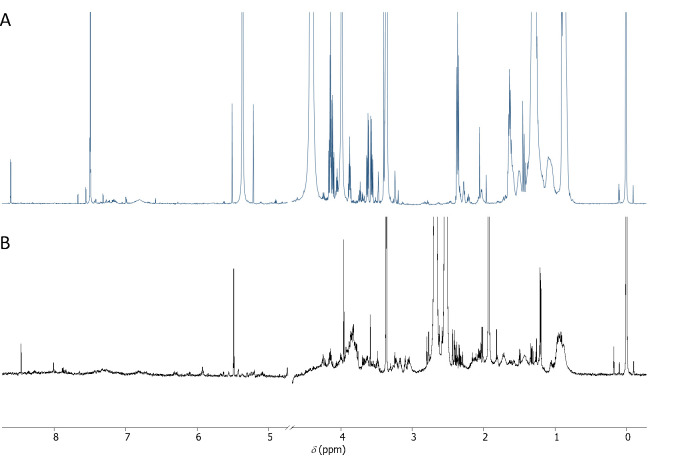
^1^H NMR spectra of photogranules metabolome. A. ^1^H NMR spectra of the lipidic phase in CDCl_3_/CD_3_OD (2/1). B. ^1^H NMR spectra of the aqueous phase in D_2_O.

In NMR, the standardized requirement for the limit of detection (LOD) and quantification (LOQ) are signal-to-noise ratios of ≥ 3 and 10, respectively [18]. In our case, most of the NMR spectra obtained had enough signals with a signal-to-noise ratio higher than 10. In the aqueous phase, on average, 120 peaks were detected per sample, and 12 ± 4 metabolites were identified per sample using the ASICS package (55 different metabolites were identified in the aqueous phase depending on the condition). The citrate signal at 2.67 ppm, coming from the RLT buffer and displayed in Figure 3, masked some information and is certainly responsible for the relatively small number of metabolites identified here (see General Note 1).

In the organic phase, around 150 peaks were detected per sample, and 17 ± 4 metabolites were identified per sample (altogether 88 different metabolites were identified in the organic phase depending on the condition). Raw NMR spectra can be found in the following database: https://doi.org/10.5281/zenodo.14383486.

**Figure 4.**
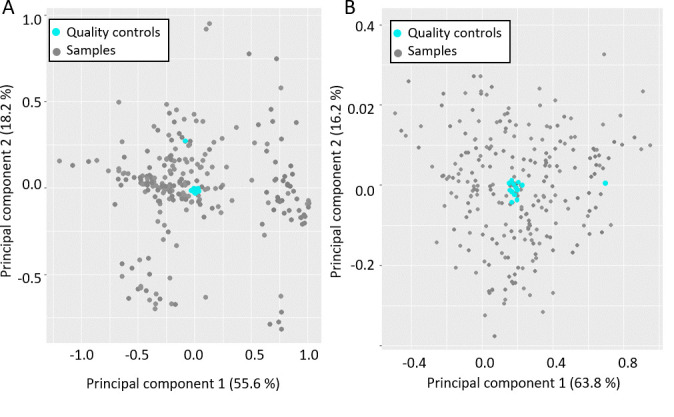
Principal component analysis (PCA) obtained from nuclear magnetic resonance (NMR) spectra. A. PCA obtained from the lipidic samples and (B) the aqueous samples. Quality controls are depicted in blue.

The quality of the analysis was controlled at multiple points for both phases to ensure that there was no drift or other introductions of errors in the results. For this, dedicated control samples (a pool of all samples, corresponding to quality controls) were analyzed repeatedly after a series of ten unknown samples. Identical spectra were expected for the quality controls in aqueous and lipid phases. Principal component analysis is one way of demonstrating that, as expected, controls for both phases were clustered together, indicating the absence of analytical derivation (Figure 4) and thus validating the analytical method.

To estimate the recovery efficiency and reproducibility of the extraction method, two conditions were tested with OPGs: (i) a condition containing OPGs in tap water (6 mg of dry biomass in 500 μL of tap water) and (ii) another one containing OPGs in tap water and acetate, which can be used as carbon source (6 mg of dry biomass in 500 μL of a tap water solution containing 400 mg acetate per liter). To assess if we could measure changes in OPG metabolic activity, samples from condition (ii) were extracted after 5 and 240 min of incubation.

**Figure 5.**
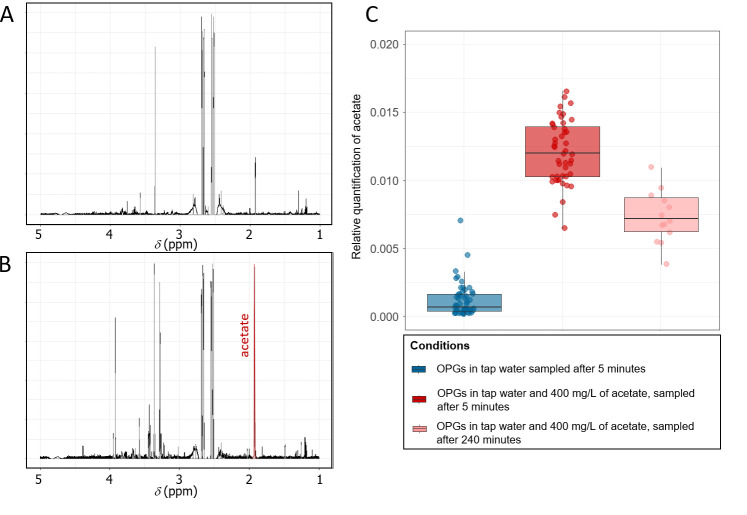
Assessment of recovery efficiency and reproducibility of the extraction method using acetate. A. ^1^H nuclear magnetic resonance (NMR) spectrum (D_2_O) of an aqueous phase extracted from oxygenic photogranule (OPG) samples without acetate input. B. ^1^H NMR spectrum (D_2_O) of an aqueous phase extracted from an OPG sample with acetate input. C. Boxplot showing the relative quantification of acetate found in samples without acetate (51 replicates, blue box), after the addition of acetate (45 replicates, red box), and after 240 min of incubation with acetate (14 replicates, pink box).

As shown in Figure 5, the extraction method coupled to the identification method with ASICS enabled the identification and recovery of acetate in NMR spectra. Furthermore, as shown in Figure 5, a relative quantification carried out on 96 samples with and without acetate clearly showed a significant difference (p-value < 0.05) between the two conditions, demonstrating the reproducibility and recovery efficiency of the extraction method. Additionally, we could also detect a significant difference in acetate relative abundance between samples taken after 5 min of incubation and samples taken after 240 min, demonstrating the metabolic activity of photogranules and the capacity of the extraction method to monitor metabolic changes.

**B. RNA quality control**

As mentioned earlier, to ensure successful the required ribodepletion for metatranscriptomics experiments, RNA concentration must be higher than 100 ng/μL. After checking that extracted RNA was pure enough, estimated by the measure of the absorbance ratios at wavelengths 260/230 nm and 260/280 nm, which should be higher than 1.8, RNA concentrations were determined using a Qubit 4 fluorometer.

After the co-extraction of 200 samples, using the described method, RNA concentration was on average 174 ± 40 ng/μL. Among the 200 samples, 186 samples had RNA concentrations higher than 100 ng/μL.

To assess the integrity of RNA after DNase treatment, RNA integrity number (RIN) values were assessed using Qubit 4 fluorometer. The higher the RIN value, the better the RNA integrity will be. In our case, we considered only samples with a RIN greater than 7. Among the 200 co-extracted samples, 181 met this quality criterion. Environmental conditions such as light and input of acetate did not influence RIN values or RNA concentrations.

C. Mass range for method validity (minimum quantity of biomaterial)

Preliminary tests aiming at optimizing the method were carried out on samples containing 20 OPGs (approximately 3 mg of dry biomass) and 50 OPGs (approximately 6 mg of dry biomass).

With 20 OPGs, the resulting NMR spectra were of poorer quality and insufficient for metabolomics analysis. For example, only six metabolites were detected in the aqueous phase extracted from 20 OPGs vs. an average of 12 for 50 OPGs. Similarly, in the organic phase, eight metabolites were identified with 20 OPGs vs. an average of 17 for 50 photogranules.

Furthermore, as mentioned above, 50 OPGs were sufficient to obtain RNA of good quality to later perform metatranscriptomics analysis (with 20 OPGs, the quantity was not sufficient).

## General notes and troubleshooting

**General notes**

1. The buffer RLT was analyzed by ^1^H NMR during the optimization of the protocol. It was shown to contain citrate at 2.67 ppm. The strong citrate signal of the buffer masks part of the NMR signal. The RLT buffer is an essential component of the RNeasy Mini Kit for RNA extraction, which contains guanidinium thiocyanate, a powerful chaotropic agent that denatures proteins and nucleases. Its purpose is to ensure effective cell lysis and prevent enzymatic degradation of RNA. The RLT buffer also facilitates the efficient binding of RNA to the silica membrane in the kit’s spin column, a crucial step for purifying RNA by separating it from other cellular components. The buffer cannot be readily replaced with an alternative when using this kit.

**Troubleshooting**

**Table:** 

Problem or issue	Likely cause(s)	Possible solution(s)
No metabolites were extracted/they underwent degradation	OPG metabolome was not frozen correctly.	Freeze the OPG metabolome in liquid nitrogen.
	The cold chain was interrupted.	Keep the samples on dry ice during the transfer between rooms or labs and directly put them into a -80 °C freezer.
	The protocol was performed by one person only, resulting in too long lag times.	Metabolite and RNA extractions are best carried out by two people to avoid lag times after steps common to both extractions.
	Extraction times between the first and last samples in a series were not identical.	In the case of high-throughput extraction, be careful to produce small series (eight samples per series).
No RNA was extracted/it was degraded	The cold chain was broken when adding the RLT buffer and/or during the grinding steps.	Use an ice bed during these steps to maintain the OPGs at a low temperature.
	RNA extraction was performed at the wrong temperature.	RNA extraction must be carried out at room temperature to avoid damaging the RNA kit columns after the addition of 70% ethanol (procedures D8a).
RNA is contaminated by DNA	No optimal DNase treatment.	Perform a new DNase digestion on the RNA eluate. Alternatively, use the RNeasy Plus Mini kit (Qiagen, catalog number: 74134), which contains a supplemental step with gDNA Eliminator Spin Columns.
Part of the ^1^H NMR spectra is masked by citrate	Citrate, an ingredient of the RLT buffer, remains in the aqueous phase after metabolome extraction.	If the citrate peak contains an essential part of the spectra, an alternative RNA extraction kit should be tested. For example, the TRIzol^TM^ Plus RNA Purification kit containing TRIzol^TM^ buffer (or phenol-guanidine reagent) could be an option.
